# Activation of PKA *in cell* requires higher concentration of cAMP than *in vitro*: implications for compartmentalization of cAMP signalling

**DOI:** 10.1038/s41598-017-13021-y

**Published:** 2017-10-26

**Authors:** Andreas Koschinski, Manuela Zaccolo

**Affiliations:** Department of Physiology, Anatomy and Genetics and BHF Centre of Research Excellence, University of Oxford, Oxford, UK

## Abstract

cAMP is a ubiquitous second messenger responsible for the cellular effects of multiple hormones and neurotransmitters via activation of its main effector, protein kinase A (PKA). Multiple studies have shown that the basal concentration of cAMP in several cell types is about 1 μM. This value is well above the reported concentration of cAMP required to half-maximally activate PKA, which measures in the 100–300 nM range. Several hypotheses have been suggested to explain this apparent discrepancy including inaccurate measurements of intracellular free cAMP, inaccurate measurement of the apparent activation constant of PKA or shielding of PKA from bulk cytosolic cAMP via localization of the enzyme to microdomains with lower basal cAMP concentration. However, direct experimental evidence in support of any of these models is limited and a firm conclusion is missing. In this study we use multiple FRET-based reporters for the detection of cAMP and PKA activity in intact cells and we establish that the sensitivity of PKA to cAMP is almost twenty times lower when measured *in cell* than when measured *in vitro*. Our findings have important implications for the understanding of compartmentalized cAMP signalling.

## Introduction

The prototypical intracellular second messenger cAMP controls a wide range of cellular processes in response to a variety of hormones, neurotransmitters and drugs^1^. The signalling pathway typically involves activation of a G_s_ coupled receptor (GPCR) at the plasma membrane and subsequent activation of adenylyl cyclase, which converts ATP into cAMP. Cells often express multiple GPCRs that signal via cAMP and the main effector of cAMP, the ubiquitous PKA, is a highly promiscuous enzyme that can phosphorylate multiple targets in each individual cell. Work in the past twenty years has firmly established that for cAMP to mediate specific effects in response to different extracellular stimuli, compartmentalization of the molecular components of the signalling pathway and differential local control of cAMP concentration are required^2,3^. Compartmentalization occurs at different levels and often involves the formation of multiprotein complexes (signalosomes) where the GPCR, the effector PKA, PKA target proteins and regulatory components, such as phosphatases and the cAMP degrading phosphodiesterases (PDEs), are organized locally by multiscaffolding proteins known as A kinase anchoring proteins (AKAPs)^4^. PDEs, by degrading cAMP locally, determine whether the PKA present in a specific multiprotein complex is activated or not and therefore dictate, at any given time, which signalosome is engaged in a signalling event^5^.

Although the model of compartmentalized cAMP signalling is now widely accepted and the details of the local subcellular organization and regulation of individual signalosomes are continuously uncovered, there are aspects of cAMP signalling that remain incompletely understood. One fundamental open question concerns the activation of PKA. Multiple studies find that in a variety of cells, including cardiac myocytes, the intracellular concentration of cAMP in unstimulated conditions is around 1 μM^6–9^. However, the EC_50_ of cAMP binding to the regulatory subunit of PKA has been reported to be in the 100–300 nM range^10,11^. Based on these values PKA should be fully activated under resting conditions and hormones acting via cAMP would not be able to trigger any PKA-dependent response. This is clearly in contrast with common observations and a number of hypotheses have been put forward to explain such inconsistency. In early studies the finding that the amount of cAMP in tissue homogenates is the same in control and hormone-treated samples, and apparently sufficient to fully activate PKA, led to the hypothesis that the majority of cAMP must be bound or sequestered and that the metabolically active second messenger is only a small fraction of the total amount^12^. More recently, intracellular free cAMP has been directly measured using FRET-based reporters and micromolar basal cAMP has been confirmed in a variety of cell types. An alternative hypothesis put forward is that the apparent activation constant value for PKA determined *in vitro* is an underestimation due to the vast excess of cAMP over pure enzyme normally used in the artificial *in vitro* set up^6^. Compartmentalization of PKA in domains with significantly lower basal cAMP than in the bulk cytosol has also been suggested^13^. Direct experimental evidence in support of any of these models is, however, very scarce. In this study we set out to resolve the apparent discrepancy between basal cAMP levels and PKA activation threshold using direct *in cell* determination of cAMP concentrations and PKA activation.

## Results

### Determination of free cAMP concentration in the bulk cytosol of intact cells

**Figure 1:**
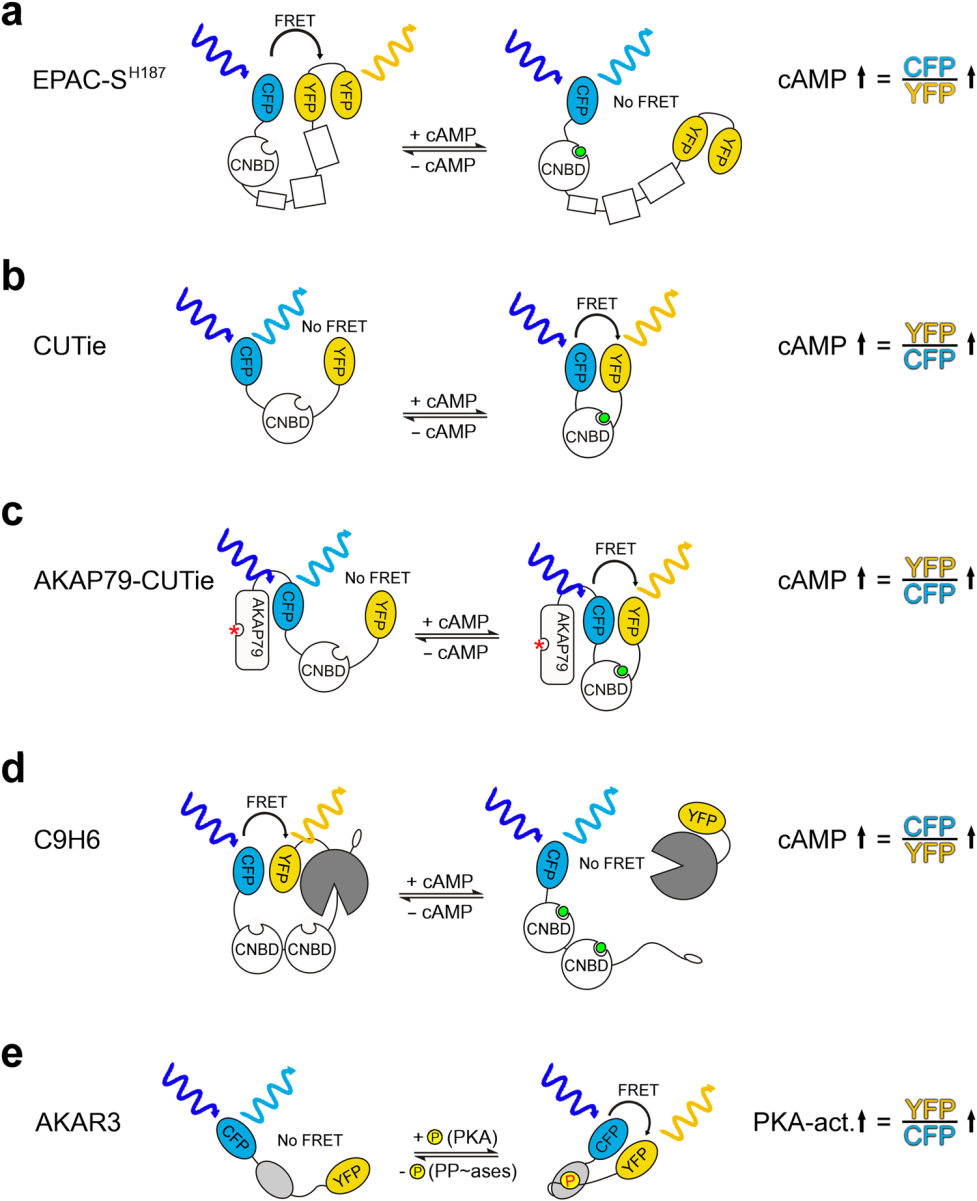
FRET sensors used in this study. Schematic representation of the different sensors illustrating the working principle. EPAC-S^H187^ and C9H6 are “loss of FRET” sensors and show decreasing FRET with increasing cAMP concentrations. CUTie, AKAP-CUTie and AKAR3 are “gain of FRET” sensors, showing increased FRET with increasing cAMP concentrations. CNBD = cyclic nucleotide binding domain, cAMP is shown in green, red star indicates the PKA anchoring site. Throughout this study the change in fluorescence intensity ratio (FRET change) is calculated so that an increase in cAMP or PKA activity corresponds to an increase in the ratio value (indicated on the right).

We first considered the possibility that previously published values for intracellular basal cAMP may be inaccurate due to methodological limitations. Earlier quantification protocols using assays that measure total cAMP in cell lysates or tissue homogenates^8^ may significantly overestimate the amount of free cAMP available to trigger PKA activation. Although free cAMP in intact cells has recently been measured using FRET-based reporters^9,14^, the protocols used relied on the assumption that the dynamic range and/or affinity of the reporter is the same in the intact cell as measured *in vitro* ^9,14^. We recently developed a calibration protocol for FRET-based reporters that uses microinfusion of known concentrations of cAMP in intact cells via a patch pipette^15^. This approach, which results in homogeneous intracellular distribution of cAMP (Supplementary Figure S1) gives the opportunity to control parameters such as ionic composition, ionic strength and pH in the patch pipette and to match them closely to physiological intracellular values (see materials and methods). Using this approach, concentration-dependency curves can be generated without any assumption based on data acquired *in vitro*. With this method the x-crossing of the cAMP concentration-dependency curve (zero FRET change) indicates the equivalence between the micro-infused cAMP concentration and the intracellular basal cAMP. To exclude that the FRET measurements may be influenced by artificial fluorescence changes (for example secondary to cAMP-dependent variations in the intracellular ionic composition that may affect fluorophore emission), we compared dose-dependency curves of two different FRET reporters, CUTie^16^ and EPAC-S^H187^. cAMP binding to these two sensors results in an opposite change in FRET (see Fig. 1a,b). We calculated cAMP changes as CFP emission/YFP emission for EPAC-S^H187^ and YFP emission/CFP emission for CUTie. Therefore, any artificial change in fluorescence occurring during the measurements would affect the ratio value determined with these sensors in an opposite manner.

**Figure 2:**
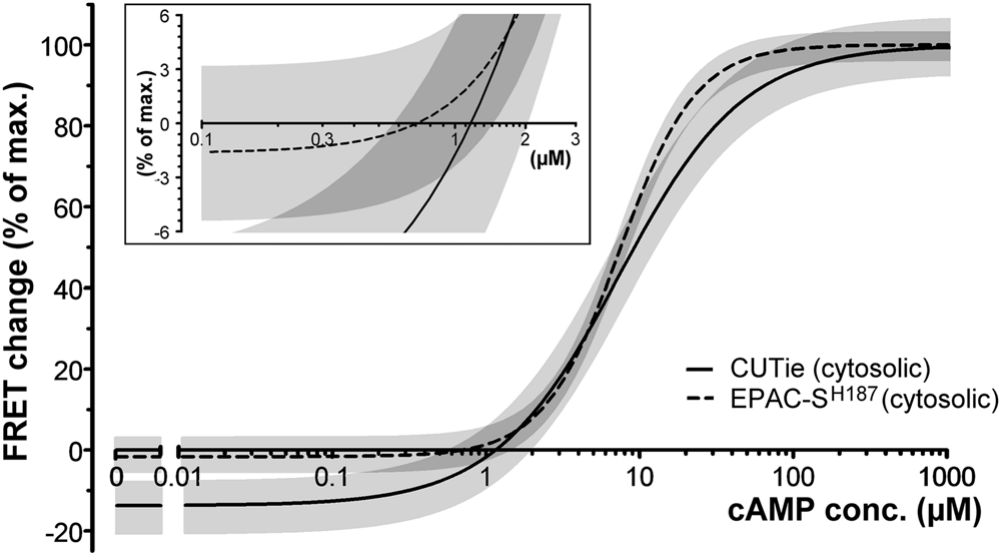
*In cell* determination of basal intracellular cAMP concentration. cAMP-FRET concentration-dependency curves generated by microinfusion of known cAMP concentrations in CHO cells stably expressing the cytosolic cAMP FRET-reporters CUTie or EPAC-S^H187^. The calculated x-crossings are 0.71 µM for EPAC-S^H187^ and 1.14 µM for CUTie. Inset shows the x-crossings in more detail. Best fit values were: −13.73% (bottom), 100% (top), 1.071 (Hill coefficient), 7.385 (EC_50_) for CUTie, and −1.650% (bottom), 100% (top), 1.750 (Hill-coefficient), 7.406 (EC_50_) for EPAC-S^H187^. Light grey shaded areas represent 95% confidence intervals and dark grey areas show the overlap. For curves including individual concentration data points see Supplementary Figure S3a,b).

As illustrated in Fig. 2, our measurements show that the x-crossing of the concentration dependency curve is around 1 μM cAMP for both CUTie and EPAC-S^H187^. To exclude the possibility that the microinfusion approach may trigger the synthesis of cAMP and affect basal cAMP levels, we measured FRET changes in saponin permealised, EPAC-S^H187^ expressing CHO cells to allow free diffusion of known concentrations of bath applied cAMP and found matching results (Supplementary Figure S2). Our *in cell* determination of basal free cAMP is therefore in agreement with previously reported values.

### Determination of basal cAMP levels at AKAP79

**Figure 3:**
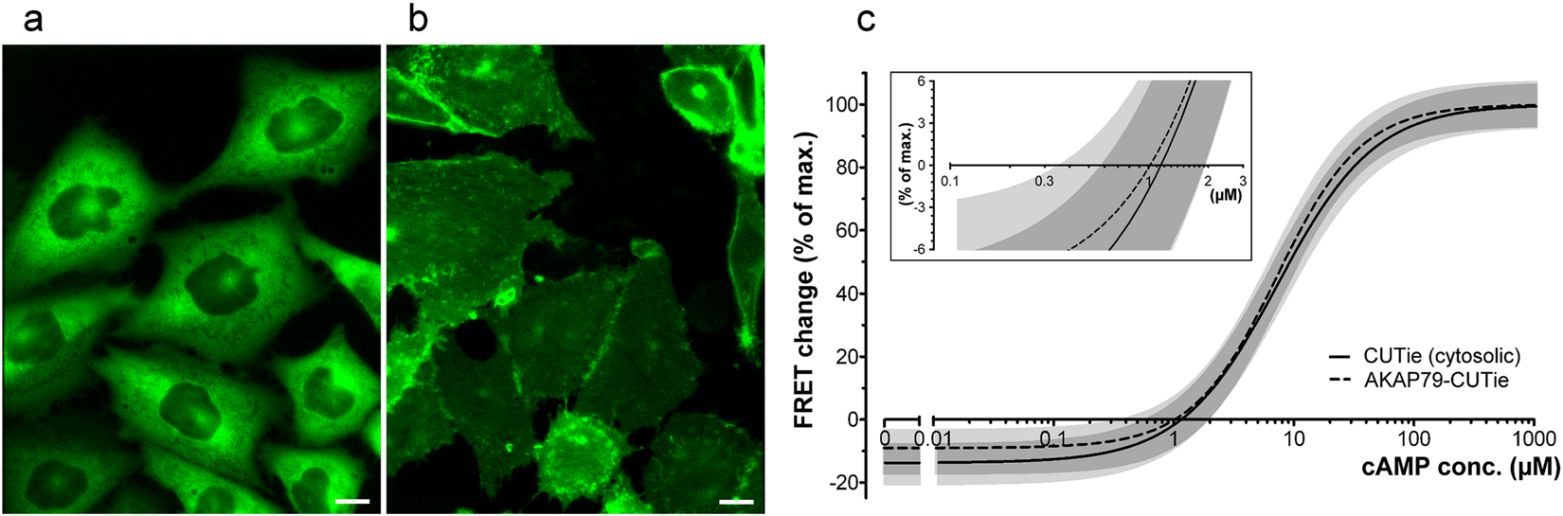
Comparison of the cAMP levels detected by cytosolic and targeted CUTie sensors. Images of (**a)** CHO-cells stably expressing cytosolic CUTie and (**b)** membrane targeted AKAP79-CUTie showing the sensor localization. Scale bars are 10 µm. (**c)** cAMP-FRET concentration-dependency curves generated by microinfusion of known cAMP concentrations in CHO cells stably expressing CUTie or AKAP79-CUTie. Inset shows a magnification of the x-crossings of the curves. Calculated x-crossing for the targeted AKAP 79-CUTie was 1.01 µM. Best fit values for AKAP 79-CUTie were: −9.080% (bottom), 100% (top), 1.230 (Hill-coefficient), 7.179 µM (EC_50_). For CUTie values refer to Fig. 2. Light grey shaded areas represent the 95% confidence intervals, dark grey areas show the overlap. For curves including individual concentration data points see Supplementary Figure S3b,c.

Based on computational studies the hypothesis has been put forward that PKA localises within microdomains where the basal concentration of cAMP may be maintained at significantly lower levels than in the bulk cytosol, thus protecting PKA from activation in the absence of extracellular stimuli^13^. To test this hypothesis we used the sensor AKAP79-CUTie^16^ (Fig. 1c). In this variant, the sensor is genetically fused to AKAP79^17^, a prototypical scaffolding protein that anchors PKA in a signalosome at the plasma membrane. When expressed in CHO cells, AKAP79-CUTie shows the expected localization (Fig. 3b), unlike CUTie which is homogeneously distributed in the cytosol (Fig. 3a). We previously demonstrated that when fused to the cAMP reporter AKAP79 maintains the ability to anchor PKA^18^ and that AKAP79-CUTie is incorporated into the correct signalosome^16^. Therefore, the cAMP concentration detected by this sensor is the level experienced by a PKA subset localized to a defined microdomain. When we microinfused CHO cells stably expressing AKAP79-CUTie with known concentrations of cAMP to generate a cAMP-FRET concentration-dependency curve for this sensor we found that the calculated x-crossing again is around 1 µM (Fig. 3c), indicating that PKA anchored to AKAP79 at the plasma membrane experiences the same basal cAMP concentration as found in the bulk cytosol.

### *In cell* determination of the activation threshold of overexpressed PKA

**Figure 4:**
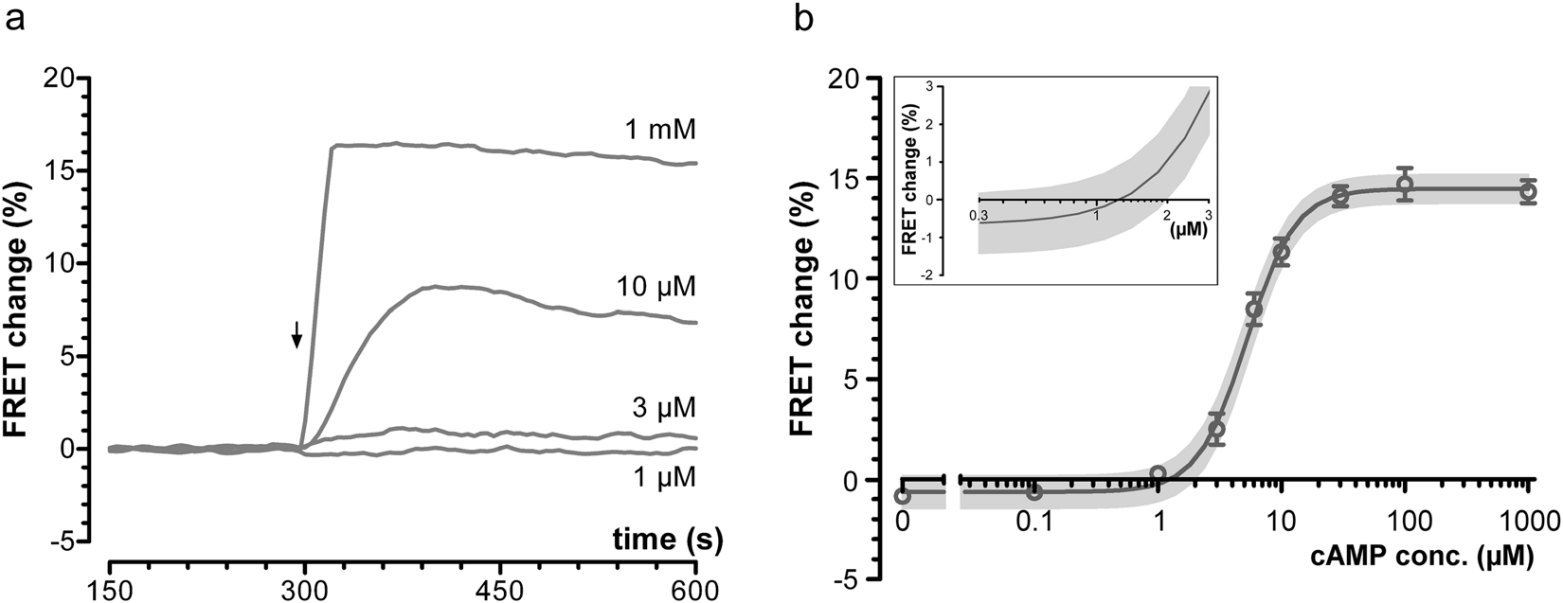
*In cell* determination of the apparent activation constant of overexpressed PKA. (**a)** Representative kinetics of FRET change recorded in CHO cells stably expressing the PKA-based sensor C9H6 and microinfused with different cAMP concentrations (as indicated). The arrow shows establishment of the whole-cell configuration and start of microinfusion. (**b**) Concentration-dependency curve for C9H6. N ≥ 3 independent experiments for each concentration. Inset shows a magnification of the x-crossing of the curve. The calculated x-crossing is at 1.26 µM. Best fit values were: −0.657% (bottom), 14.47% (top), 2.191 (Hill-coefficient), 5.231 µM (EC_50_). Gray shaded areas represent the 95% confidence interval for the curve.

We next investigated the extent of PKA activation at basal cAMP. For this we used the C9H6 FRET reporter^19^ which is based on the regulatory (R) and catalytic (C) subunits of PKA fused to YFP and CFP, respectively (Fig. 1d). With increasing cAMP concentrations, this sensor dissociates in free R-CFP and C-YFP subunits leading to loss of FRET signal. We previously demonstrated that fusion of the PKA subunits to CFP and YFP does not affect cAMP binding to the holoenzyme or its enzymatic activity^11^. When measured *in vitro* the apparent activation constant (EC_50_ value) for C9H6 was 0.3 μM^20^, in agreement with *in vitro* values reported for native PKA. However, when CHO cells stably expressing C9H6 were microinfused with 1 µM cAMP, a concentration that, based on the *in vitro* EC_50_ value, should almost maximally activate PKA, no FRET change was detected (Fig. 4a). Microinfusion of 3 µM cAMP induced only minor FRET changes, whereas 10 µM cAMP induced a significant increase. Microinfusion of 1 mM cAMP saturated the sensor. The concentration-dependency curve shows that, when measured *in cell*, the apparent activation constant of C9H6 is 5.2 μM, almost 20 times higher than the value measured *in vitro* (Fig. 4b).

### Evaluation of endogenous PKA activation threshold

**Figure 5:**
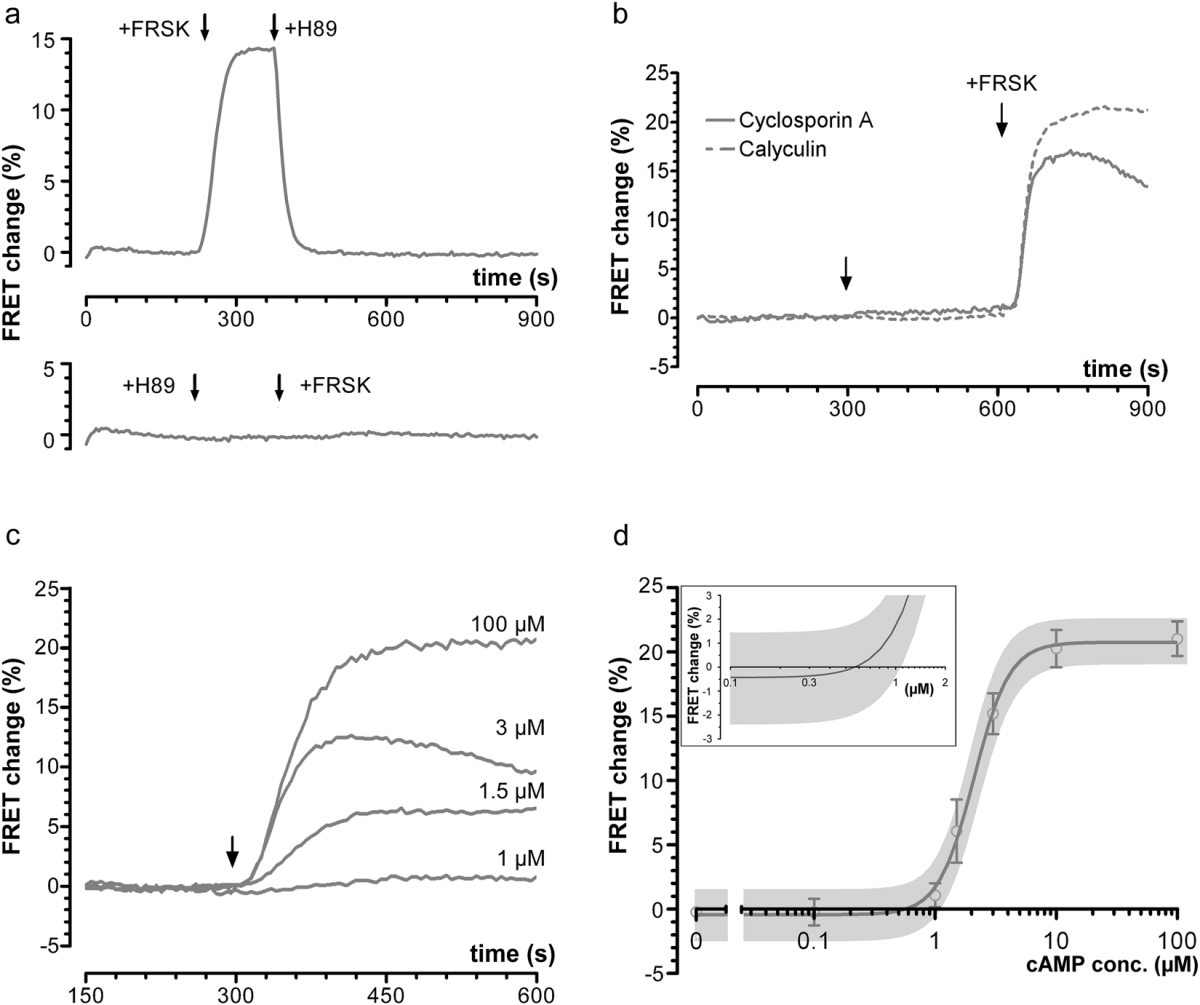
*In cell* determination of endogenous PKA activity. (**a)** upper panel: trace showing the avarage FRET change recorded in CHO cells stably expressing AKAR3 and treated with forskolin (1 µM) prior to the addition of H89 (10 µM). Lower panel: Effect of H89 (10 µM) on the FRET signal recorded at basal conditions. Subsequent application of 10 µM forskolin shows no FRET change confirming complete blockage of PKA activity. N = 31 cells for both traces. (**b)** Average kinetics of FRET change detected in CHO cells expressing AKAR3 and treated with Calyculin (10 nM,) or Cyclosporin A (200 nM), as indicated. Forskolin (10 µM) was applied to confirm that the AKAR sensor is responsive to PKA phosphorylation. Arrows indicate time of application. N ≥ 27 cells for both traces. Traces in **a** and **b** are representative for at least 3 independent experiments. (**c)** Representative kinetics of FRET changes recorded in CHO cells stably expressing the PKA-activity reporter AKAR3. The arrow indicates establishment of the whole-cell configuration and start of microinfusion. cAMP concentrations in the pipette are indicated close to the respective curve. (**d)** Concentration-dependency curve for the AKAR3-sensor. Inset shows a magnification of the x-crossing of the curve. The calculated x-crossing is at 0.57 µM. Best fit values were: −0.4348% (bottom), 20.74% (top), 2.971 (Hill-coefficient), 2.086 µM (EC_50_). Grey shaded areas represent the 95% confidence interval for the curve. N ≥ 3 independent experiments for each concentration.

Previous *in vitro* measurements showed that the apparent activation constant of PKA is influenced by the concentration of the enzyme used in the assay, with increasing concentrations of cAMP being necessary to activate increasing concentrations of the purified enzyme to the same extent^6^. In our experimental conditions we expect the concentration of overexpressed C9H6 to exceed that of endogenous PKA. To assess whether this may explain the unexpectedly high EC_50_ values found for C9H6 we used AKAR3 (Fig. 1e), a FRET-based PKA activity reporter, to measure the activity of endogenous PKA in intact cells. AKAR3 is activated by PKA-mediated phosphorylation and de-activated by dephosphorylation (see Fig. 1e). Therefore, a significant basal activity of endogenous PKA would be expected to result in a detectable change in the FRET signal reported by AKAR3 upon cell treatment with the PKA inhibitor H89. We found that while application of 10 µM H89 completely reversed the AKAR3 FRET change induced by activation of adenylyl cyclases with 1 µM forskolin (Fig. 5a), there was no detectable change in the AKAR3 FRET signal when H89 was applied in otherwise untreated cells (Fig. 5a). These results indicate that there is no significant activity of endogenous PKA at basal levels of cAMP.

To exclude that the activity of phosphatases may mask any basal PKA-dependent phosphorylation of AKAR3 we treated CHO cells expressing AKAR3 with phosphatase inhibitors. Treatment with either calyculin A (10 nM) or cyclosporin A (200 nM) revealed no significant dephosphorylation of AKAR3 (Fig. 5b).

To assess the activation threshold of endogenous PKA we next generated a concentration-dependency curve for AKAR3 phosphorylation using the cAMP microinfusion method. As shown in Fig. 5c,d, AKAR3 shows a steep cAMP concentration-dependency and the cAMP concentration required for half-maximal PKA-dependent phosphorylation of AKAR3 is about 2.1 µM. Extrapolating from this curve, the level of AKAR3 phosphorylation at 1 µM cAMP can be estimated to be about 10% of maximum, confirming minimal activity of endogenous PKA.

**Figure 6:**
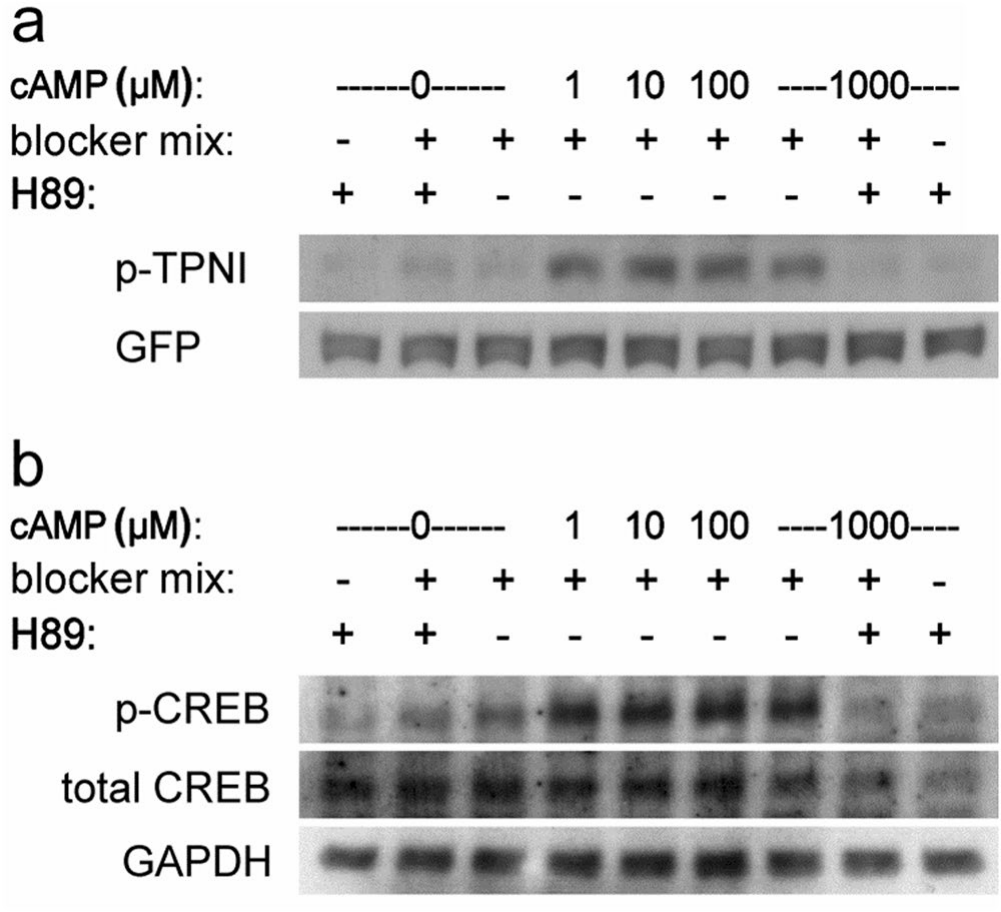
PKA activity in CHO cells lysates. (**a**) Western blotting of lysates from CHO cells expressing a GFP-tagged version of the PKA target protein troponin I and probed with a phospho-troponin I specific antibody (p-TPNI) and a GFP specific antibody for total troponin I. (**b**) Western blotting of endogenous protein in lysates from CHO cells probed with phospho-CREB antibody (p-CREB) and total CREB antibodies. Blocker mix consists of phosphatase and PDE inhibitors. Blots are representative of at least 3 independent experiments. Shown are the relevant bands. Full-size blots are presented in Supplementary Figure S4.

In support of a significant difference in the activation threshold of PKA *in vitro* and *in cell*, when we assessed PKA-dependent phosphorylation in lysates obtained from CHO cells we found that addition of 1 µM cAMP to the lysate was sufficient to maximally phosphorylate the PKA targets troponin I and CREB (Fig. 6).

**Figure 7:**
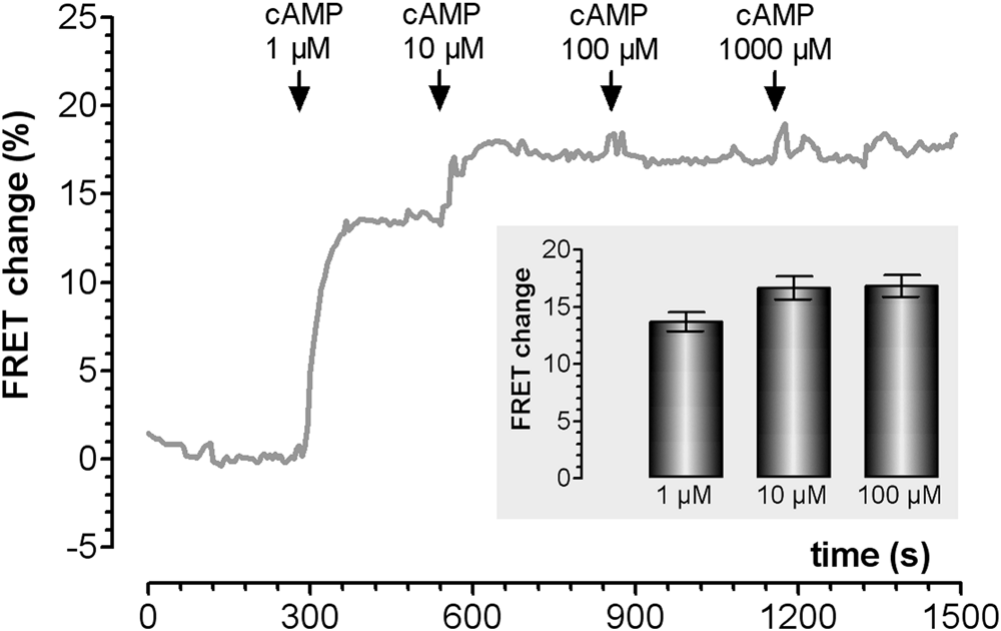
Effect of different concentrations of bath-applied cAMP on the FRET signal generated by C9H6 PKA in cell lysates. Representative trace showing the time course of FRET change detected in a cell lysates from CHO cells stably expressing the sensor C9H6 PKA and challenged with different concentrations of cAMP as indicated. Insert: mean FRET change (± SEM) of 3 independent experiments at the indicated concentrations.

In line with the above findings, when we measured the FRET change generated by the PKA-based FRET sensor C9H6 in a cell lysate rather than in intact cells, we found that application of 1 µM cAMP results in 80% maximal FRET change. Further application of 10 µM cAMP only slightly increased the FRET signal and no additional FRET change was observed at 100 or 1000 µM cAMP (Fig. 7).

## Discussion

In this study we demonstrate that the activation threshold of PKA in the intact intracellular environment is significantly higher (at least one order of magnitude) than previously thought based on *in vitro* measurements. Our findings resolve the incongruity of basal cAMP concentration being sufficient to almost maximally activate PKA.

The possibility that considerable differences may exist between the control of enzyme reaction rates *in vitro* and *in vivo* has long been appreciated^21–23^. This has been ascribed, at least in part, to the fact that *in vitro* measurements are usually conducted with a large excess of substrate over enzyme, a condition that does not necessarily apply to enzymes in their intracellular environment. For example, *in vitro* measurements showed that while 0.3 μM cAMP is necessary to half-maximally activate 0.009 μM PKA, five-fold higher concentration of cAMP (about 1.5 μM) is necessary to activate to the same extent 0.15 μM PKA, a concentration of the enzyme that is closer to the estimated intracellular concentration of PKA (0.23 μM in skeletal muscle)^6^.

Here, by directly measuring cAMP levels and PKA activation in intact cells we directly assessed the apparent activation constant of PKA *in cell* and found that it is about twenty-fold higher than the value previously determined *in vitro*. A number of other factors, in addition to enzyme concentration, may contribute to the inaccuracy of the *in vitro* measurements, including artificially low ionic strength or low pH, both known to affect PKA activation^24^, as well as lack of physiological cofactors that may be present in the cellular environment but missing in the *in vitro* settings^6^. Although our study concerns PKA, it is reasonable to expect that a similar fault may apply to *in vitro* measurements reported for other enzymes.

Our findings have important implications for the understanding of cAMP signalling and the model of cAMP/PKA compartmentalised signalling. A large body of evidence supports a role of PDEs in limiting cAMP homogeneous diffusion inside the cell. Several studies from different laboratories demonstrate that inhibition of PDEs disrupts the boundaries between cAMP pools and results in equilibration of cAMP levels across the cell, indicating that the ability of PDEs to degrade cAMP contributes to the compartmentalization of the second messenger^25–30^. One criticism of this model has been that when taking into account the apparent activation constant of PKA as determined *in vitro* (in the 0.1–0.3 μM range) and the reported K_M_ and V_max_ values for PDEs (see Table 1), it is difficult to envisage how PDEs may be able to maintain the concentration of cAMP below the activation threshold of PKA even at basal cAMP levels, let alone contribute to compartmentalization of the cAMP response to hormonal stimulation, when the levels of cAMP significantly increase^13,31^. Based on these considerations, it has been argued that PDEs cannot dictate what subset of PKA is activated in response to a given stimulus, simply because their activity is inadequate to reduce cAMP levels below the PKA activation threshold.

**Figure 8:**
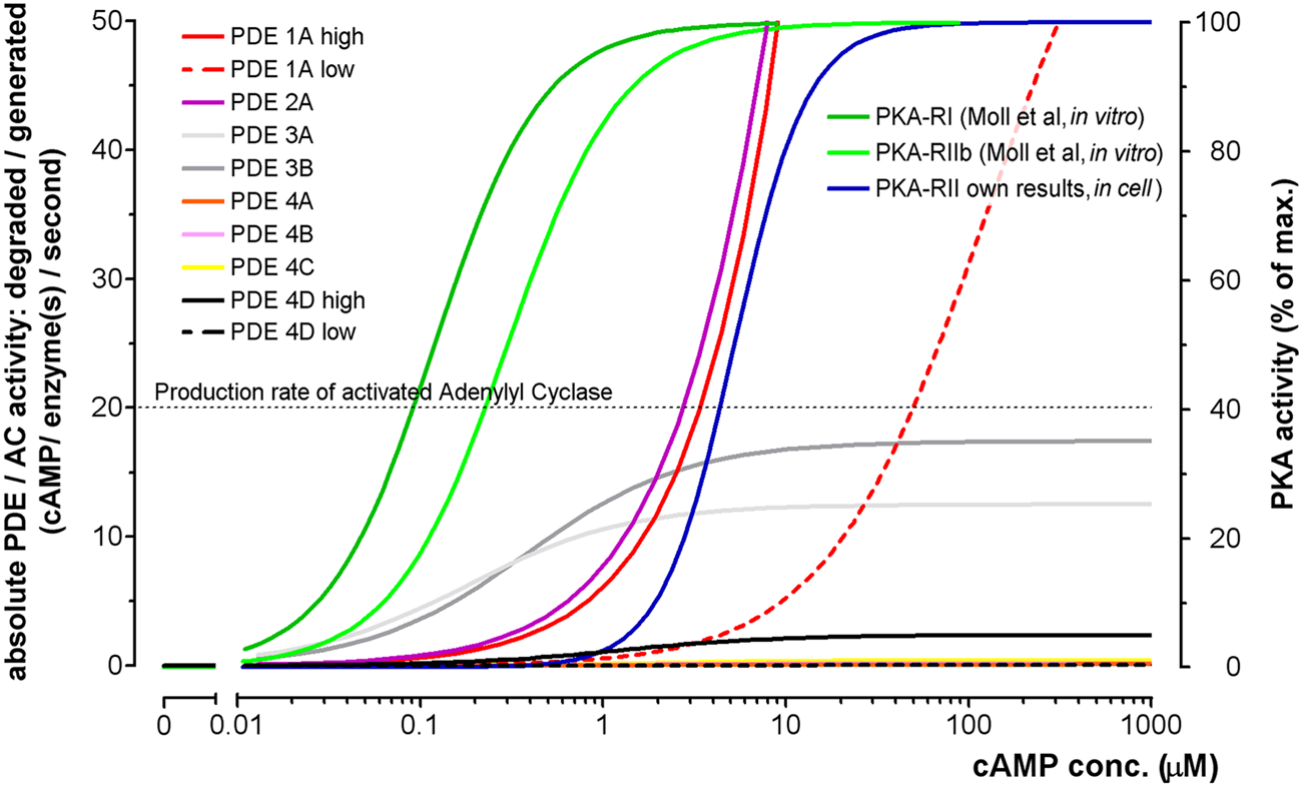
Comparison of the concentration-dependent activities of PKA (right ordinate) and the absolute activities of selected PDEs (left ordinate). “High” and “low” denote high and low affinity states of the respective PDE. Note that the activity of all PDE4 isoforms, except PDE4D in its high affinity state, are too low to be distinguishable at this scaling factor. All PDE curves are calculated according to the corresponding K_M_ values reported in the literature (see Table 1) and assuming, as a first approximation, that K_d_ ≈ K_M_ ≅ EC_50_. The respective absolute activity was calculated from V_max_ and used to set the “top” of the curves (see Table 1). “Bottom” was set to zero. As cAMP-degradation by PDEs is a simple dual molecule reaction without cooperativity a maximal Hill-coefficient of 1 was assumed. *In vitro* PKA curves (green) are recalculated according to^20^, *in cell* PKA activity (blue) is calculated according to values found in this study. Also shown is the production rate of adenylyl cyclase^39^. All activities are calculated as number of cAMP-molecules degraded/generated in one second by one PDE/AC molecule.

The problem is illustrated in Fig. 8 which shows the concentration dependency curves for selected PDEs and for PKA calculated from *in vitro* values found in the literature (for conversion and standardization of values see Materials and Methods). Synthesis rate of cAMP by adenylyl cyclases is also shown. The graph shows that, for example, only at a cAMP concentration of about 2 µM would one molecule of PDE3A or PDE2A be able to degrade cAMP rapidly enough to compensate for the production of cAMP by one active AC. Comparison of these curves shows clearly that none of the PDEs would be able to degrade cAMP fast enough to maintain the level of cAMP below the activation threshold of PKA as determined *in vitro*. If we consider an intracellular concentration of PKA of 0.23 µM (as measured in some cells types^6^), to achieve effective degradation of cAMP a concentration of PDEs several order of magnitude higher would be required. However, estimates based on literature values and our own measurements indicate that the overall PDE concentration in cardiac myocytes is in the range of 0.22 µM (for estimation see Methods), insufficient to effectively degrade cAMP to the required level. In contrast, PDEs would be able to maintain basal cAMP below the activation threshold of PKA as determined *in cell* (shown in blue in Fig. 8), even when cAMP synthesis is activated.

The high apparent PKA activation constant determined *in cell* has significant impact on the quantitative evaluation of cAMP/PKA signal transduction and it will be important to include this value in future computational studies that model cAMP signalling. In this respect, it is interesting to note that some in silico analyses^32^, including our recent study^16^, already make the assumption that the affinity of cAMP for PKA is about ten times lower than the *in vitro* reported values. Our findings also indicate that efforts should be made in the future to determine *in cell* the enzyme reaction rates of other components of the cAMP/PKA signalling pathway. For example, it would be of key importance to establish kinetic parameters for the PDEs in the intracellular environment. At the same time, caution should be used when drawing conclusions on the basis of parameters constrained by experimental data acquired *in vitro*.

## Materials and Methods

### Cell culture and generation of stable cell lines

Chinese hamster ovary cells (CHO) were grown in Ham’s-F12 supplemented with 10% fetal bovine serum FBS (Gibco, Art. No. 10270, lots 41F0374K and 41G5630K), 100 units/mL penicillin, 100 µg/mL streptomycin, and 2 mM glutamine at 37° Celsius and 5% CO_2_. Stable cell lines were generated using TransIT®-LT1 transfection reagent (MIR 2300, Mirus) and plasmidic DNA according to the manufacturer’s guidelines. Transfected cells were then grown in selection medium containing 800 µg/ml G418, and in the case of the C9H6 PKA-based sensor additionally 300 µg/ml Zeocin™ (InvivoGen). Clones were selected using the infinite dilution method. AKAR3^33^ and EPAC-S^H187^ ^34^ FRET sensors were kind gifts from Jin Zhang (University of California, San Diego) and Kees Jalink (The Netherlands Cancer Institute, Amsterdam), respectively.

### In cell calibration procedure by microinfusion

CHO-cells stably expressing the FRET sensors were patch-clamped and simultaneously observed under FRET-excitation. After establishment of a tight seal between cell-membrane and patch-pipette (“Gigaseal”) the membrane under the pipette-tip was ruptured and the whole-cell configuration was established. This configuration provides direct access from the pipette to the cytoplasm and cAMP can either diffuse from the pipette into the cell or vice versa, depending on the cAMP-concentration in the patch-pipette solution. FRET-ratio changes were monitored for different cAMP concentrations in the patch pipette and computed into concentration-dependency curves. Seal- and whole-cell resistances were monitored in parallel to ensure a permanently tight seal between pipette and cell-membrane. Seal procedure was performed under DIC illumination. Illumination was switched to fluorescence after a seal was successfully established. Holding potential was 0 mV, with a test-pulse of −10 mV. Pipettes were pulled from borosilicat glass (Hilgenberg) with a Sutter-Puller P-2000 (Sutter Instruments) and had a tip diameter of about 2 µm. Pipette resistance was in the range of 5–10 MΩ. Seal resistances typically were in the range of several Gigaohm, whole-cell resistances for CHO-cells were typically in between 0.5 to 1 Gigaohm. Pipette solutions (“intracellular buffer”) contained 20 mM NaCl, 140 mM K-Glutamate, 2 mM MgCl_2_, 0.00404 mM CaCl_2_, 0.1 mM BAPTA (yielding a calculated free Ca^2+^-concentration of 10 nM, low buffered) and 10 mM HEPES. For measurements using CUTie-based sensors^16^ K-Glutamate was replaced by 140 mM KCl. For AKAR3 measurements the solutions were supplemented with 2 mM ATP and 0.3 mM GTP. All solutions were adjusted with KOH according to the measured intracellular pH of the cells (here pH 7.64) and supplemented with various cAMP-Na-concentrations. The extracellular solution contained 140 mM NaCl, 3 mM KCl 3, 2 mM MgCl_2_, 2 mM CaCl_2_, 15 mM Glucose, 10 mM HEPES and was adjusted with NaOH to pH 7.2. Electrophysiological data were acquired with a Cairn Optopatch patch-clamp amplifier (Cairn Research) controlled by WinWCP software (John Dempster, University of Strathclyde).

FRET measurements were performed with a Nikon Eclipse FN-1, equipped with an Opto-LED fluorescent light source (Cairn-Research), a Dual-View beam splitter (Optical Insights) and a CoolSnap HQ^2^ camera (Photometrix). All images were acquired with a 40x/0.8 numerical aperture, long distance water dipping objective (Nikon). Excitation wavelength was 436 ± 25 nm, excitation/emission dichroic was 455 nm long pass. Emission light was split by a 505 nm longpass dichroic and filtered at 480 ± 15 nm for CFP emission and 535 ± 20 nm for YFP emission. Acquisition and analysis was performed using Optofluor (Cairn Research). All FRET measurements were background-subtracted and, if necessary, corrected for baseline drifts. FRET changes were determined at steady state indicated by a plateau of the ratio change.

As especially the YFP fluorophores are prone to quenching effects due to pH or Cl^−^, for a correct determination of basal cAMP-values it was crucial to determine the intracellular pH and the influence of different ionic conditions on the FRET signal to correct for artificial shifts. For determination of the intracellular pH, cells expressing the FRET reporter were placed in a “high K^+^” extracellular buffer (KCl: 140 mM, NaCl: 4 mM, MgCl_2_: 2 mM, CaCl_2_: 2 mM, Glucose: 15 mM, HEPES: 10 mM), adjusted to different pHs (range 6.8 to 8.0) with KOH or HCl. Nigericin (10 µM) and Valinomycin (5 µM) were added to permeabilize the membranes to H^+^and K^+^^35^ and the change in FRET signal was recorded over time. pH-dependency curves were generated and a pH of 7.55 to 7.64 (depending on the different cell clones) was identified as the pH value that did not affect the FRET signal, indicating that this pH reflects the mean intracellular pH of the CHO cellline (adapted from^35^). All intracellular buffers were subsequently matched to the pH of the cell clone under study.

For some measurements a KCl-based intracellular patch-clamp buffer containing 162 mM Cl^−^ was used. Whereas the normal intracellular Cl^−^-concentration of CHO-cells should be around 22 mM^36^,we performed FRET-experiments microinfusing CHO-cells with 22 or 162 mM Cl^−^, respectively, at various cAMP-concentrations to control for possible quenching effects. These experiments showed that at high Cl^−^ the cAMP-dependent FRET signal is shifted to 1.7% more negative values (mean of 4 different cAMP concentrations). FRET changes recorded in conditions of high Cl^−^ were therefore corrected for this shift. The saponin permeabilisation protocol was as in^37^ except for that the “intracellular buffer” was used in the bath.

All microinfusion/pH- experiments were carried out at 24–28 °C. All mathematical corrections and analyses were performed with Excel™, Open Office and GraphPad Prism™. Best fit curves were generated using the built in fit function for sigmoidal dose response curves (variable slope, no constraints) of Graph Pad Prism™. All values are means ± SEM unless stated otherwise. Graphical editing/layout of figures and Western blot scans were performed with Micrografx Picture Publisher 8 and 10 (Micrografx Inc.). Where necessary, image processing was carried out according to the digital image integrity rules and standards.

### Western blotting

Wild type CHO cells or CHO cells stably expressing TPNI-CUTie^16^ were lysed in ice-cold intracellular buffer with added protease blocker (cOmplete mini, EDTA-free, Roche), by 15 strokes through a 0.4 mm syringe needle. The lysate was aliquoted in pre-cooled tubes, shock-frozen and transferred to −80 °C until used for the assay. A reaction mix was prepared containing intracellular buffer supplemented with 2 mM Na-ATP, 0.3 mM Na-GTP, cAMP and, where appropriate, with phosphatase inhibitors (Calyculin, 50 nM, Cyclosporin, 200 nM), phosphodiesterase inhibitor (IBMX, 100 µM) and PKA-inhibitor (H89, 30 µM). All operations were carried out on ice with chilled solutions. The phosphorylation reaction was started by injection of lysate (20 µg total protein as determined by Bradford assay) into the reaction mix. Final volume was 50 µl. Reaction time was 3 or 10 minutes at 37 °C. The reaction was stopped by shock-freezing the mix in a dry ice/ethanol bath (−72 °C, approx. freezing time 10 s). The samples (8 µg total protein) were loaded on a 4–12% Bis-Tris gels (Novex™, Life Technologies) and transferred onto nitrocellulose membranes (0.45 µm, Amersham™ Protron™, GE Healthcare Ltd.) using Bolt transfer buffer (Novex™, Life Technologies). Proteins were probed with anti p-TPNI (phospho S22 + S23) (ABCAM, AB58545), anti p-CREB (Phospho S133) (ABCAM, AB32096), anti GFP (B2) (Santa Cruz, SC9996), anti CREB (48H2) (Cell Signaling Technology^®^, #9197), anti GAPDH (H12) (Santa Cruz, SC166574). Secondary antibodies were anti Mouse IgG (Promega, W4028) and Goat anti Rabbit IgG (Santa Cruz, SC2004). Blocking agent was 5% PhosphoBLOCKER™ (Cell Biolabs, Inc.) for initial blocking and primary antibodies or 1% for secondary antibodies. Membranes were stripped using RESTORE™ Western Blot stripping buffer (Thermo Scientific). p-TPNI, p-CREB and CREB were imaged with Super Signal® West Dura (Thermo Fisher Scientific, #34075), GFP and GAPDH with Pierce® ECL Western Blot Substrate (Thermo Fisher Scientific, #32106) on Amersham™ Hyperfilm™ ECL (GE Healthcare Ltd.).

### ***In vitro*** FRET measurements

Lysates of CHO cells stably expressing the C9H6 PKA sensor were prepared as described for Western-blot experiments and imaged with the same setup as described for FRET experiments, except that we used a self-made microcuvette and optimized the imaging settings to compensate for the weak fluorescence intensity of the lysate (camera-binnig 8, Gain 2, 1 s exposure time and 5 Watt excitation energy). All values are background-subtracted considering the dark-noise as background value.

## Normalization of data from the literature

### Conversion of V_max_ into absolute activity

To allow comparison of the cAMP-degrading activity of different PDE’s and to relate PDE activity to the production of cAMP by ACs we calculated the “absolute activity” as molecule(s) cAMP per molecule enzyme per second.

**Table 1:** K_M_, V_max_ and calculated absolute activity values for selected PDE’s: Table 1.

PDE-Isoform	Approx. mol. weight (KDa)	K_M_ for cAMP (µM)	V_max_ (µmol × min^−1^ × mg^−1^)	Calculated absolute. activity: molecules cAMP × molecule enzyme^−1^ × s^−1^
PDE1A	60	72.5–124	70–450	70–450
PDE1B	62	10–24	10	10.3
PDE1C	72	0.3–1.1	—*	—*
PDE2A	103	30	120	206
PDE3A	125	0.18	3–6	6.25–12.5
PDE3B	123	0.38	8.5	17.4
PDE4A	90	2.9–10^40^	0.058^40^	0.09
PDE4B	85	1.5–4.7	0.13	0.18
PDE4C	80	1.7	0.31	0.41
PDE4D	90	1.2–5.9	0.03–1.56	0.045–2.34
PDE8A	100	0.06^41^	0.15^41^	0.25
PDE8B	89	0.1^42^	0.23^42^	0.34

K_M_ and V_max_ values according to^43^ or as indicated by the references. V_max_ was determined with purified enzyme. Approximate molecular weights according to various sources. For calculations see Materials and Methods section. *: no available data source.

As typically V_max_ is expressed as “µmol substrate × mg enzyme^−1^ × minute^−1^” and PDEs differ in their weight significantly, we first calculated the number of molecules per mg according to equation 1, using the conversion 1 KDa ≅ 10^6^ mg.

1 $$\frac{6\cdot {10}^{23}\,{molecules}}{{M}{{W}}_{{PDE}}\,[{mg}]}={x}\,[{molecules}\,{PDE}\times {m}{{g}}^{-1}]$$

As the number of cAMP molecules (the substrate) per μmol is 6·10^−17^, it is possible to calculate the absolute enzyme-activity in the directly comparable format: “degraded molecule(s) cAMP per molecule enzyme per second” (equation 2).

2 $$\frac{{V}_{\max }[{\mu }{mol}\times {m}{{g}}^{-1}\times {60}\,{{s}}^{-1}]\times {6}{\cdot }{{10}}^{{17}}[molecules\,{cAMP}\times {\mu }{mo}{{l}}^{-1}]}{x\,[molecules\,{\rm{PDE}}\times {{\rm{mg}}}^{-1}]\,\times {60}}=absolute\,enzyme\,activity$$

The values for the molecular weight, K_M_, V_max_ and the calculated absolute activities for selected PDEs are listed in Table 1. As the activity values are derived from V_max_ they served as maximum (“top”) values to calculate the corresponding curves in Fig. 8.

### Assumptions for the conversion of K_M_ into EC_50_

The Michaelis constant K_M_ = (k_cat_ + k_r_)/ k_f_ differs from the dissociation constant K_d_ = k_r_/k_f_ regarding the influence of k_cat_. However, it might be assumed that for most enzymes K_d_ will be close to the value of K_M_, as in most cases k_cat_ is small compared to k_r_ and k_f_.

Therefore, as a first approximation, we can assume that a cAMP concentration leading to half-maximal binding will also lead to half-maximal reaction velocity (K_M_ ≈ K_d_). Thus, for PDEs K_d_ is considered as equivalent to EC_50_.

### Estimation of absolute numbers of selected components of the cAMP-signalling pathway

As some data were not available for CHO cells, we used data from neonatal cardiomyocytes for our estimations:

Volume of neonatal cardiomyocytes: 819 µm^3^ (1 day in culture) to 1532 µm^3^ (3 days in culture)^38^. As our cardiomyocytes are typically measured after 2 days in culture, we assumed 1000 µm^3^, corresponding to 1 pl (1·10^−12^ l).

Estimation of the number of PDE’s per cell and effective degradation rate: Mongillo *et al*.^11^ determined the total PDE activity of neonatal cardiomyocytes (in lysate assays) to be 102 ± 8 pmol/minute/mg protein. From our own experiments we can calculate that 1 mg protein is equivalent to about 4.285 million cells. This enables us to calculate the effective degradation rate for the overall PDE population in one cell to be 240,000 cAMP molecules per second.

If we assume that the majority of PDEs in neonatal cardiomyocytes consists of about 30% PDE3 and 60% PDE4^11^ and we assign mean degradation activities of about 10 × s^−1^ for the PDE3 fraction (6.25–12.5 for PDE3A, 17.4 for PDE3B), 1.5 × s^−1^ for the PDE4 fraction (PDE4D, 60% of the PDE4 population, turnover 0.045 for non-activated, up to 2.37 for activated, all other PDE4s only 0.09 to 0.41) and 1 × s^−1^ for all other PDEs, we can estimate that the population of PDEs consists of about 7,200 PDE3 molecules, 96,000 PDE4 molecules (thereoff 1,800–2,400 PDE4B molecules^39^) and 24,000 molecules for all other cAMP degrading PDEs. All activities are calculated from V_max_ values as shown in Table 1. This means a total number of about 130,000 PDEs per cell, equivalent to a concentration of about 0.216 µM.

Estimation of the number of PKA-molecules per cell: Beavo *et al*.^6^ calculated a concentration of up to 0.23 µM PKA in rabbit skeletal muscle cells. Assuming a similar concentration in cardiac myocytes and the cell volume to be 1 pl, this would be equivalent to about 138,000 PKA molecules per cell.

### Data availability

All datasets generated during and/or analysed during the current study are available from the corresponding author on reasonable request.

## Electronic supplementary material

Dataset 1
