# Supplementary material for: Activation of PKA in cell requires higher concentration of cAMP than in vitro: implications for compartmentalization of cAMP signalling
Source: Sci Rep. 2017 Oct 26;7:14090. doi: 10.1038/s41598-017-13021-y (PMC5658382; doi:10.1038/s41598-017-13021-y)
Supplement: Supplementary file 1 — Dataset 1 [file 41598_2017_13021_MOESM1_ESM.doc]

**Supplementary information for**

**Activation of PKA *in cell* requires higher concentration of cAMP than *in vitro*: implications for compartmentalization of cAMP signalling.**

Andreas Koschinski and Manuela Zaccolo^*^

Department of Physiology, Anatomy and Genetics and BHF Centre of Research Excellence, University of Oxford, Oxford, UK

**Supplementary Figure S1**


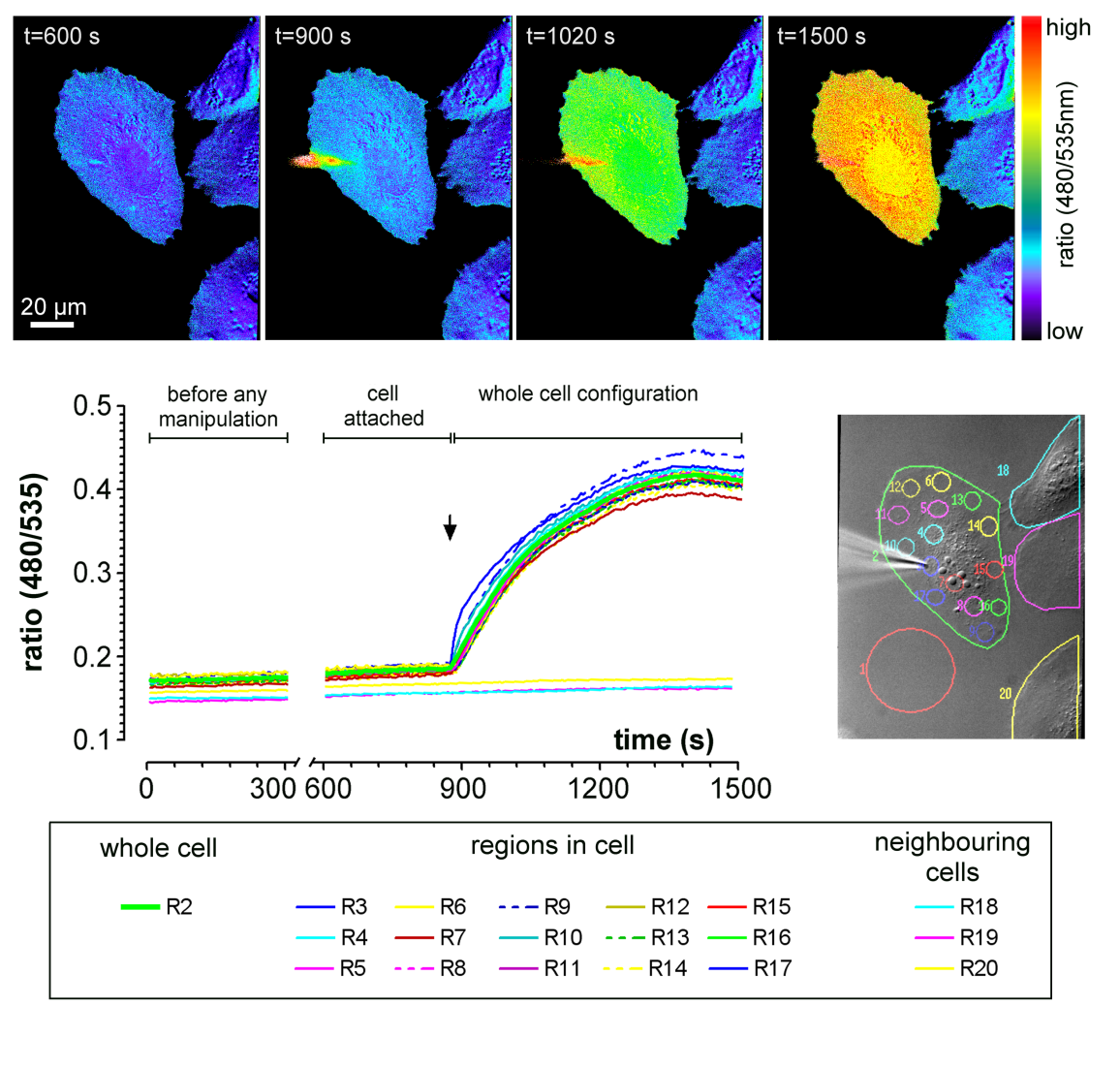


**Time-course of FRET changes on microinfusion of cAMP**:

Top panels: Pseudocolor images of the intensity ratio (cyan/yellow) of CHO cells transfected with the EPAC-S^H187^ sensor. The cell in the center was infused with 10 µM cAMP. Blue indicates the lowest cAMP concentration and red the highest. The lower left panel shows the time course of the ratio change before and after the whole- cell configuration is established. Traces are representative of 3 independent experiments. Cells were monitored for 5 minutes prior to any manipulation (left part of the curve). Then the pipette was approached and the tight seal with the cell membrane was established. The arrow indicates the time point when direct access to the cytosol was established ("whole-cell" configuration). The DIC image of the cells (lower right) shows the position of the Patch-Pipette used for the microinfusion and the location of the different regions analyzed.

**Supplementary Figure S2:**

| **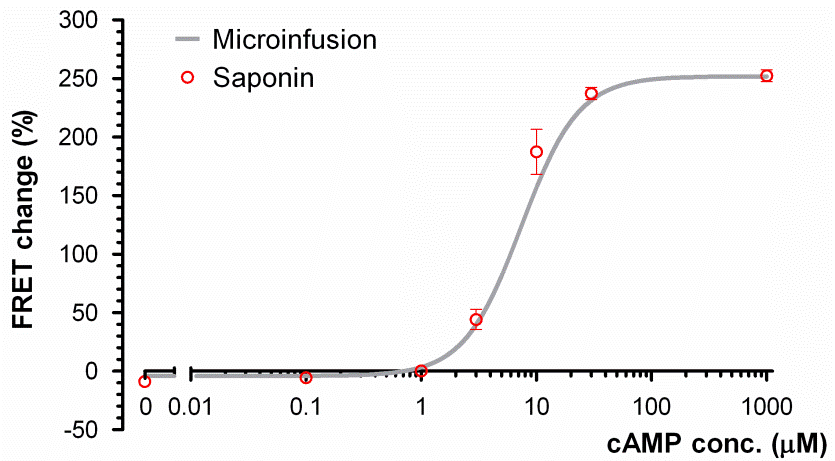** | **Comparison of FRET change induced by cAMP application via microinfusion or membrane permeabilization**: CHO cells stably transfected with the EPAC-S^H187^ FRET sensor were permeabilized with saponin (8 µg/ml) in the presence of various cAMP concentrations. The resulting FRET change after permeabilization (red circles) is compared with the curve generated by microinfusion (gray curve, Fig. 2 in main text). All experiments were performed in the same intracellular buffer as used for the microinfusion. All saponin data points are means of 8-20 cells ± SEM. |
| --- | --- |

**Supplementary Figure S3**

| **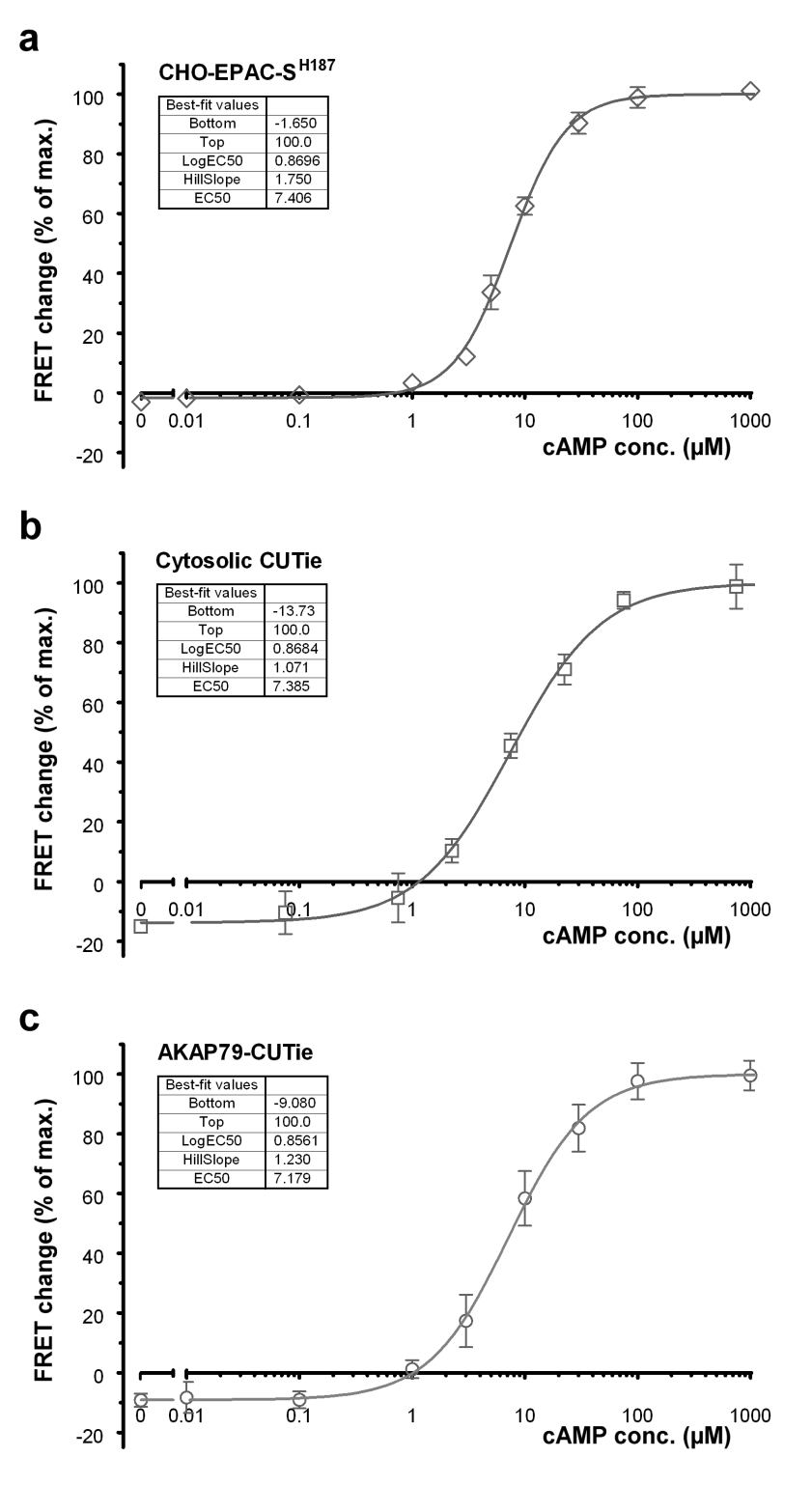** | ***In cell* cAMP-FRET dose-dependency curves** generated by microinfusion of known cAMP concentrations in CHO cells stably expressing **a):** EPAC-S^H187^, **b):** cytosolic CUTie or **c):** AKAP79-CUTie FRET sensors.  Best fit curves were generated using the built in fit function for sigmoidal dose response curves (variable slope, no constraints) of Graph Pad Prism™. Best fit values are indicated in insets. N ≥ 3 independent experiments for each concentration. All values are mean ± SEM. |
| --- | --- |

**Supplementary Figure S4**

| **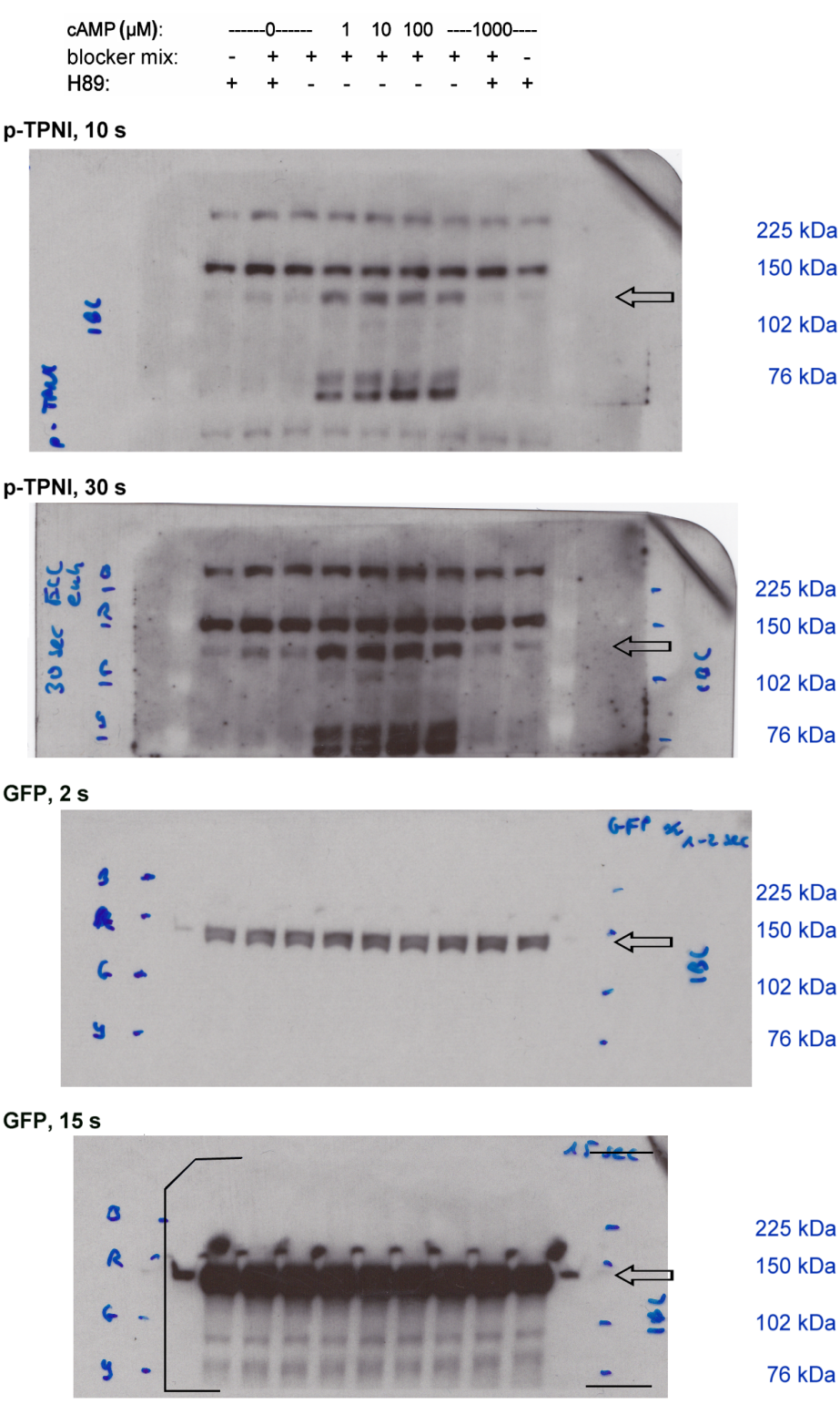** | **Original full size scans of the western blots shown in Figure 6: PKA activity in CHO cell lysates.**  **a):** Western blotting of lysates from CHO cells expressing a GFP-tagged version of the PKA target protein troponin I and probed with a phospho-troponin I specific antibody (p-TPNI) and a GFP specific antibody for total troponin I. Shown are two different exposure times for p-TPNI, of which the upper one (10 s) was shown in Fig. 6. The bands of interest are indicated by an arrow. A longer exposure time (15 s) for the GFP blot is included to provide information of the position of the bands relative to the full membrane. Borders of the membrane are indicated by black lines. |
| --- | --- |

| 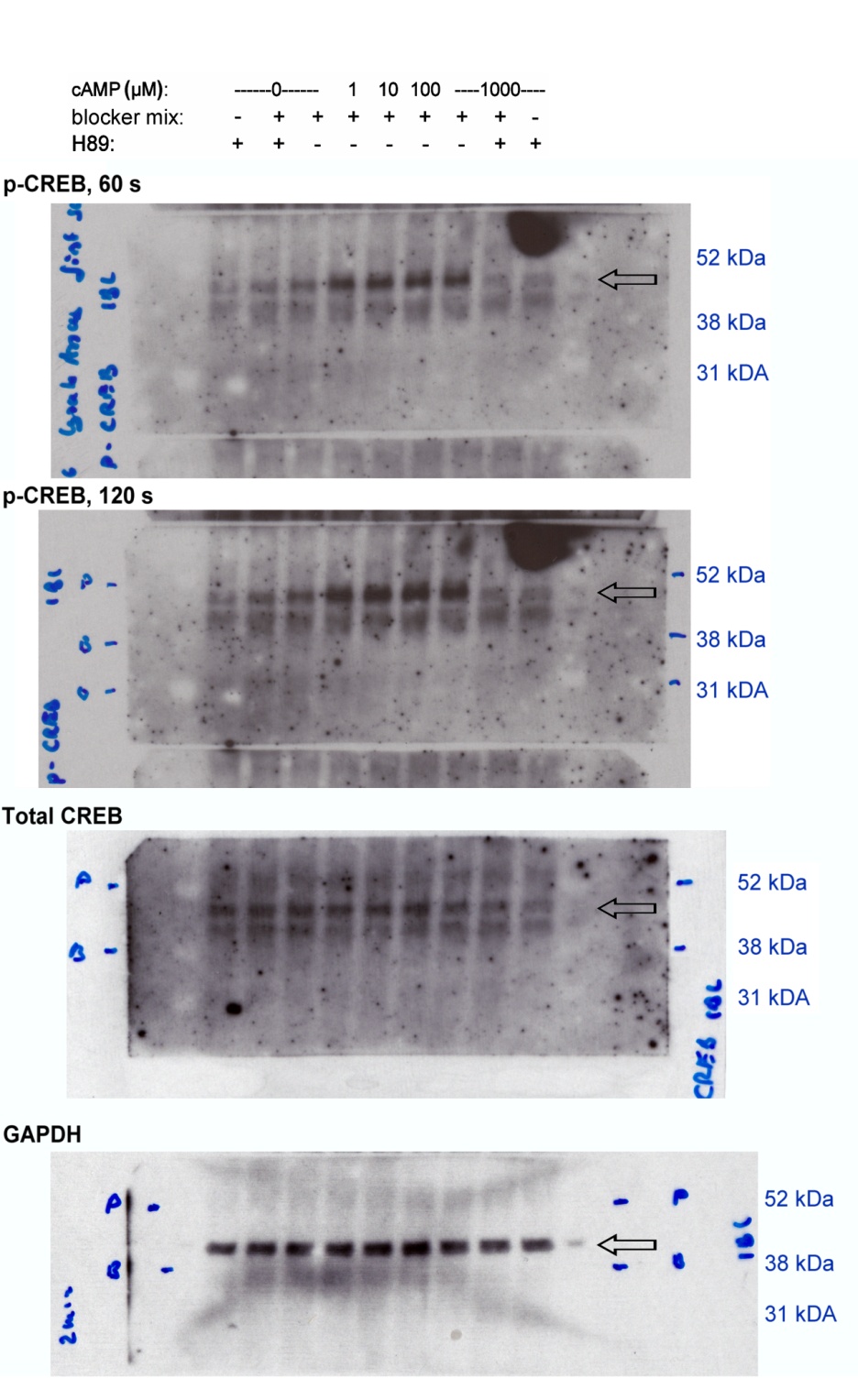 | **b):** Western blotting of endogenous protein in lysates from CHO cells probed with phospho-CREB antibody (p-CREB) and total CREB antibodies. Shown are two different exposure times for p-TPNI, of which the upper one (60 s) was shown in Fig. 6. The bands of interest are indicated by an arrow. |
| --- | --- |
